# Titration of oxygen therapy in critically ill emergency department patients: a feasibility study

**DOI:** 10.1186/s12873-018-0169-2

**Published:** 2018-06-26

**Authors:** Anna S. M. Dobbe, Renate Stolmeijer, Jan C. ter Maaten, Jack J. M. Ligtenberg

**Affiliations:** 1 Emergency Department, Internal Medicine, University Medical Center Groningen (UMCG), Hanzeplein 1, 9700 RB Groningen, The Netherlands; 2 BSc of Medicine, Faculty of Medicine, University of Groningen, Groningen, The Netherlands; 3 Emergency physician, Emergency Department, University Medical Center Groningen, Groningen, The Netherlands; 4 Internist acute medicine, Emergency physician, Emergency Department, University Medical Center Groningen, Groningen, The Netherlands

**Keywords:** Emergency medicine, Oxygen inhalation therapy, Hyperoxia, Normoxia, Hypoxia, Titration of oxygen therapy, Emergency department, Critically ill patients, Pulmonary disease, chronic obstructive, Prospective studies, Oxygen

## Abstract

**Background:**

Liberal use of oxygen in an emergency situation is common. Today, most health care professionals do not adjust the amount of oxygen given when a saturation of 100% or a PaO2 which exceeds the normal range is reached- which may result in hyperoxia. There is increasing evidence for the toxic effects of hyperoxia. Therefore, it seems justified to aim for normoxia when giving oxygen. This study evaluates whether it is feasible to aim for normoxia when giving oxygen therapy to patients at the emergency department (ED).

**Methods:**

A prospective cohort study was performed at the ED of the University Medical Center Groningen (UMCG). A protocol was developed, aiming for normoxia. During a 14 week period all patients > 18 years arriving at the ED between 8 a.m. and 23 p.m. requiring oxygen therapy registered for cardiology, internal medicine, emergency medicine and pulmonology were included. Statistical analysis was performed using student independent t-test, Mann–Whitney U-test, Fisher’s exact test or a Pearson’s chi-squared test.

**Results:**

During the study period the study protocol was followed and normoxia was obtained after 1 h at the ED in 86,4% of the patients. Patients with COPD were more at risk for not being titrated to normal oxygen levels.

**Conclusions:**

We showed that it is feasible to titrate oxygen therapy to normoxia at the ED. The study results will be used for further research assessing the potential beneficial effects of normoxia compared to hyper- or hypoxia in ED patients and for the development of guidelines.

## Background

Oxygen is one of the most widely used drugs and is applied in a wide range of medical specialities [1]. Supplemental oxygen is vital in many clinical situations; impaired oxygen delivery in critically ill patients is associated with increased mortality. Therefore, reassuring oxygen delivery has become a cornerstone in resuscitation and liberal use of supplemental oxygen in an emergency situation is common. [2] In acute medical care, toxicity of oxygen therapy is not immediately obvious. Over the past years, several studies have shown the potential harmful effects of hyperoxia, resulting in increased mortality [3–5]. Guidelines for emergency oxygen use have been developed, recommending to aim for normal or near-normal oxygen saturation for all acutely ill patients, such as the British Thoracic Society (BTS) guideline [1]. Despite these studies and guidelines, in real-life the amount of oxygen given is often not decreased if the SaO_2_ measured by pulse oximeter is 100%, or if PaO_2_ levels are high [5, 6]. Considering that the Emergency Department (ED) is the department where many critically ill patients arrive, it is important to cause no additional harm due to hyperoxia and to treat patients with the right dose of oxygen. The only study outside the ICU (in prehospital setting) aiming for normoxia had to be stopped because it appeared to be not feasible [7]. That is why we investigated if it is feasible to titrate oxygen therapy immediately after arrival at the ED. This prospective study evaluates the feasibility of a protocol aiming for normoxia in adult, critically ill patients at the ED.

## Methods

### Study population & research design

This prospective cohort study was performed at the ED of the University Medical Center Groningen, a tertiary care teaching hospital with 33.000 ED visits/year. During a 14 week period from February until May 2016, consecutive patients > 18 years arriving at the ED, registered for cardiology, internal medicine, pulmonary medicine and emergency medicine, requiring oxygen therapy (according to the judgement of the ambulance nurse, ED nurse or ED physician) were included from 8.00 h a.m. until 23.00 h p.m. The institutional review board of our hospital waived informed consent. Exclusion criteria were: intubated patients or patients requiring immediate intubation, patients with unreliable pulse oximetry recording [8] and patients using bleomycin, because high concentrations of inspired oxygen (FiO2) increases lung toxicity in these patients.

Also, patients with a known specific target saturation due to other causes at presentation were excluded, such as patients with congenital heart disease in whom saturations < 85% were normal.

**Fig. 1:**
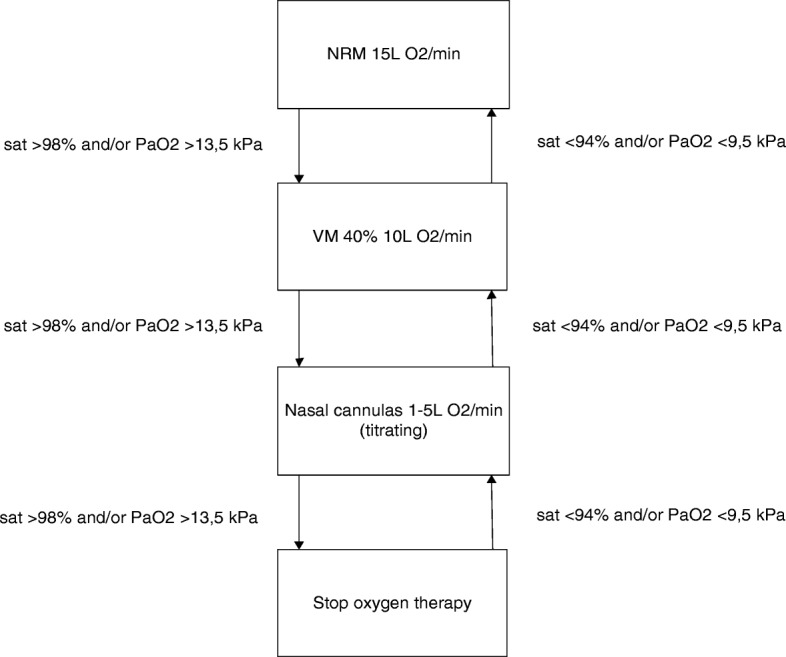
Study protocol aiming for normoxia. In severe COPD patients the same protocol was used, but saturations were different (92% instead of 98 and 90% instead of 94%)

A protocol was developed aiming for normoxia, defined as PaO_2_ 9,5–13,5 kPa (70–100 mmHg) or a corresponding oxygen saturation of 94–98%. Hyperoxia was defined - according to normal values used in our hospital - as PaO_2_ > 13.5 kPa or SaO_2_ > 98%, and hypoxia as PaO_2_ < 9.5 kPa or SaO_2_ < 94%. In consultation with our pulmonologists saturations between 90 and 92% were used to aim for normoxia in patients with known severe COPD (GOLD III or IV), in order to avoid hypercapnia, while further using the same study protocol (Fig. 1). The study protocol is shown in Fig. 1. For example, if a patient was admitted with a non-rebreathing mask (NRM) (15 L O_2_/minute, FiO_2_ = ± 0.8) and peripheral oxygen saturation (SaO_2_) immediately after arrival was ≥98%, the nurse would apply a Ventimask (VM) 40% with 10 L O_2_/minute (FiO_2_ = ± 0.4). If after 5 min, in arterial blood gas analysis PaO_2_ was still > 13.5 kPa or (peripheral) SaO_2_ was ≥98%, oxygen was titrated towards an oxygen saturation (SaO_2_) between 94 and 98%, preferably administered by a nasal oxygen cannula. If the patient was admitted with a nasal oxygen cannula and SaO_2_ was ≥94% immediately after arrival, oxygen was titrated towards a SaO_2_ between 94 and 98%. If SaO_2_ was < 94%, a VM 40% with 10 L O_2_/minute (FiO_2_ = ± 0.4) would be applied and the same procedure as described above would be followed. Patients with known Chronic Obstructive Pulmonary Disease (COPD) GOLD III of IV dropped out of the study if they became hypercapnic (PaCO_2_ > 6.0 kPa or > 45 mmHg) in the first hour after arrival. This threshold was chosen in consultation with our pulmonologists to prevent respiratory acidosis caused by relative hyperoxia lowering the respiratory rate. Oxygen saturation values were measured by non-invasive pulse oximetry [Masimo Corporation, CA, USA] and if the treating physician requested an arterial blood gas, PaO_2_ as well as oxygen saturation were used [ABL800 flex, Radiometer, Copenhagen, DK].

### Data collection

All ED nurses and physicians were educated about the purpose of the study and the protocol. Data was collected using a case report form. The values of SpO_2_ and PaO_2_ (if available) were measured at ED presentation (T_0_), after 5–10 min (T_1_), after 1 h (T_2_), and after 3 h (T_3_) together with the dose of oxygen and the method used to administer oxygen. Vital signs were measured at the same time points. When the patient was admitted to a nursing ward or ICU, the same parameters were also collected 24 h after arrival at the ED (T_4_). No advice was given about aiming for normoxia at the nursing ward or ICU. The Modified Early Warning Score (MEWS) was calculated on arrival and after 3 h.

### Outcome parameters

The primary outcome parameter was whether the protocol was followed and whether normoxia was obtained. This was based on the measurements made by the pulse oxymeter, to enchance clinical applicability. The protocol was followed when during the time the patient was admitted at the ED (between arrival at the ED and 3 h after arrival at the ED) normoxia was achieved. If only at the final measurement (3 h after arrival at the ED) the SaO_2_ was too high or too low, but the first three measurements were in the normal range, it was also concluded that the protocol was followed.

### Statistical analysis

Baseline characteristics and results are presented as mean and standard deviation (SD) when normal distribution was assumed by means of a Kolmogorov–Smirnov test. Skewed variables are presented as median and interquartile range. Categorical variables are presented as frequency and percentage. For independent continuous variables with a normal distribution, student independent t-test was used. Other continuous variables were compared using the Mann–Whitney U-test. Categorical variables were compared using Fisher’s exact test or a Pearson’s chi-squared test. A significance level of *p* < 0.05 was used.

## Results

### Study population

**Fig. 2:**
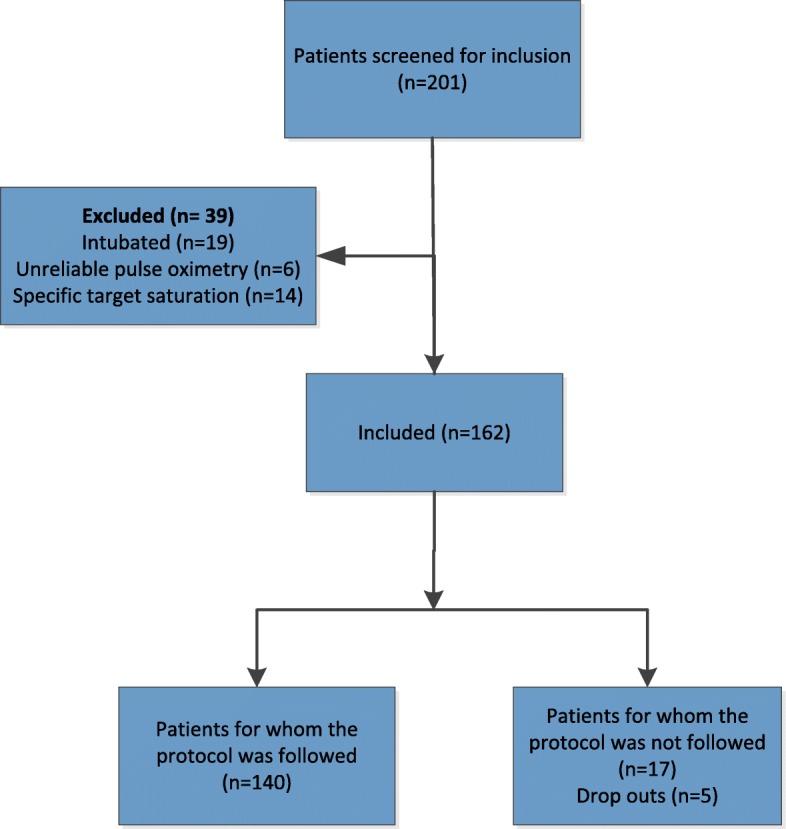
Flowchart of patient inclusion and exclusion

In the study period 201 patients were screened for inclusion; 162 patients were included. The flowchart of patient inclusion and exclusion is shown in Fig. 2. In 140 patients the protocol was followed and normoxia successfully achieved (86,4%) within 1 h after arrival at the ED. In 7 normoxic patients multiple blood gases were additionally sampled during ED admission, of these 6 were normoxic measuring both SaO2 and PaO2. In 22 patients the protocol was not followed, of which there were 5 known COPD patients who dropped out due the development of hypercapnia in the first hour at the ED. There were different reasons why the protocol was not followed in the other 17 patients. The most important observation was that most of these patients suffered from COPD. It appeared that in some COPD patients the threating physician seemed reluctant to follow the protocol, resulting in not reducing oxygen therapy in patients with severe COPD (*n* = 11) or too low oxygen saturations in patients with mild COPD (*n* = 3). For other patients we could not find a specific reason for not following the protocol.

The baseline characteristics of the 162 study patients are shown in Table 1. Significantly more patients had severe COPD (GOLD III or IV) in the group in which the protocol was not followed (*P* < 0.001). This group also received significantly more often oxygen before ED arrival.

**Table 1:** Patient characteristics at ED arrival

Characteristics		Patients in which the protocol was followed (*n* = 140)	Patients in which the protocol was not followed (*n* = 22)	Total (n = 162)	*P*-value
Male sex [n (%)]		82 (59)	11 (50)	93 (57)	0.45
Age (years)		68 ± 13	68 ± 12	68 ± 13	0.78
Prehospital oxygen	Yes [n (%)]	94 (67)	18 (82)	112 (69)	0.16
Within target at arrival*	Yes [n (%)]	56 (60)	1 (6)	57 (51)	0.00
PaO_2_ (kPa)		9 (8–12)	8 (8–11)	9 (8–12)	0.53
Systolic blood pressure (mmHg)		125 (107–141)	137 (119–165)	126 (109–145)	0.04
Diastolic blood pressure (mmHg)		76 (65–89)	76 (65–103)	76 (65–89)	0.49
Heart rate (beats/min)		88 (75–108)	95 (88–120)	91 (78–110)	0.06
Respiratory rate (breaths/min)		21 (18–25)	22 (20–27)	21 (18–25)	0.17
GCS [1–15]**		15 (15–15)	15 (15–15)	15 (15–15)	0.24
MEWS		4 (3–5)	4 (3–6)	4 (3–5)	0.27
Smoking [n (%)]		30 (21)	8 (36)	38 (24)	0.12
Cardiac condition [n (%)]		57 (41)	10 (46)	67 (41)	0.68
COPD GOLD I/II [n (%)]		27 (19)	3 (14)	30 (19)	0.53
COPD GOLD III/IV [n (%)]		14 (10)	11(50)	25 (15)	0.00
Other pulmonary condition [n (%)]		19 (14)	5 (23)	24 (15)	0.26
Neurological condition [n (%)]		1 (1)	1 (5)	2 (1)	0.13

*Within target saturation on arrival at the ED with prehospital oxygen

**GCS, Glasgow Coma Scale

### Diagnosis at discharge

An overview of the diagnosis at discharge from the ED for the total study population is shown in Table 2. Some patients were discharged with more than one diagnosis. In the group in which the protocol was not followed more patients were discharged with a COPD exacerbation (*P* = 0.03) or with other pulmonary conditions (*P* = 0.04) compared to the group in which the protocol was followed. Other diagnoses at discharge were not significantly different between the patient groups.

**Table 2:** Diagnosis at discharge from the ED

Diagnosis at discharge from ED	Protocol was followed [n (%)]	Protocol was not followed [n (%)]	Total [n (%)]	*P*-value
Pneumonia	30 (21)	4 (18)	34 (21)	1.00
Infection	28 (20)	6 (27)	34 (21)	0.41
Heart failure	22 (16)	4 (18)	26 (16)	0.76
Other	23 (16)	1 (5)	24 (15)	0.20
Sepsis	22 (16)	2 (9)	24 (15)	0.54
COPD exacerbation	14 (10)	8 (36)	22 (14)	0.03
Cardiac condition other	17 (12)	0 (0)	17 (11)	0.13
Pulmonary condition other	10 (7)	5 (23)	15 (9)	0.04
Malignancy	11 (8)	1 (5)	12 (7)	1.00
Neurological	6 (4)	1 (5)	7 (4)	1.00

### Prehospital oxygen

The median PaO_2_ of patients arriving with prehospital oxygen was 9.5 (8.1–11.8) kPa. There is an effect of the method of oxygen delivery on causing hyperoxia at T_0._ In patients arriving with a NRM, 50% was hyperoxic at T_0_ compared to 18% of the patients with oxygen administered via a nasal cannula (*P* < 0.001).

### Oxygen saturation at the ED and after 24 h

**Fig. 3:**
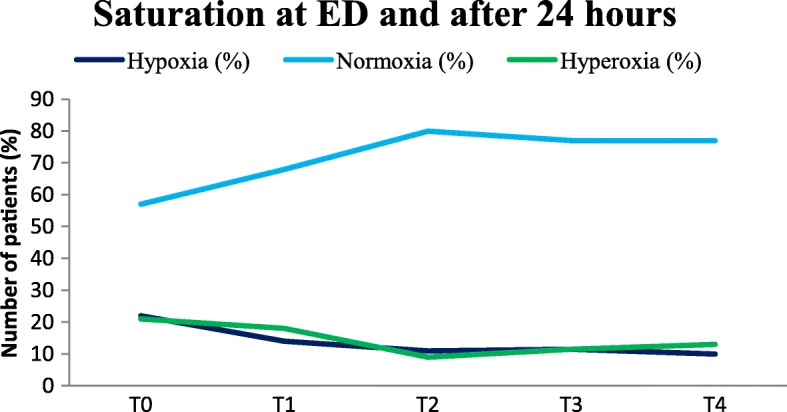
Course of oxygen saturation for all study patients (*n* = 162). On arrival at the ED (*T* = 0), after 10 min (*T* = 1), after 1 h (*T* = 2), after 3 h (*T* = 3) and after 24 h (*T* = 4)

Figure 3 shows the course of the oxygen saturation at the ED and 24 h after arrival at the ED for all included patients. When patients stopped receiving oxygen therapy and their saturation was > 94% or > 90% in severe COPD patients, they were considered to be normoxic. In some patients their oxygen saturation fluctuated during ED admission, resulting in shifting between hypoxia, normoxia and hyperoxia at the different time points. From the figure it becomes clear that most patients were in the normoxia range after 1 h at the ED. In the first 10 min (T_0_-T_1_) it was more difficult to attain normoxia, maybe due to the amount of oxygen treatment in the ambulance or due to the number of actions that have to be performed when a patient arrives at the ED. Some patients were normoxic at first but at T_3_ they became hypoxic or hyperoxic, probably because they were checked less frequently. When patients were admitted to the ICU or nursing ward, most patients stayed in the normoxia range.

### Oxygen saturation of severe COPD patients

**Fig. 4:**
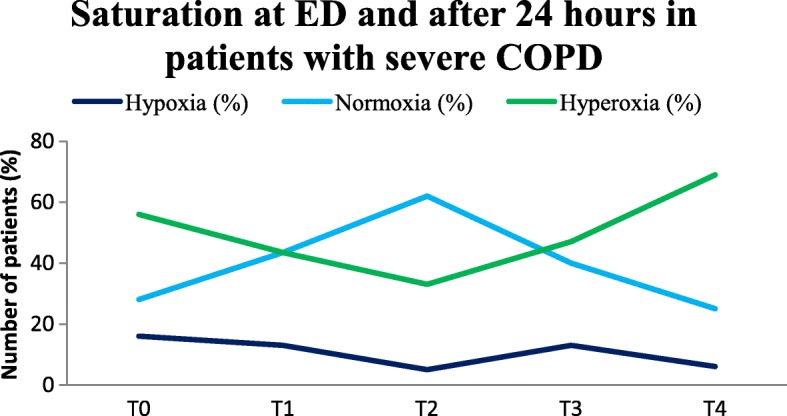
Oxygen saturation COPD GOLD III and IV patients (*n* = 25). On arrival at the ED (T = 0), after 10 min (T = 1), after 1 h (T = 2), after 3 h (T = 3) en after 24 h (T = 4)

In patients with severe COPD the protocol was more often not followed. For this reason, we made a separate graph for this subgroup, shown in Fig. 4.

Figure 4 shows that almost half of the severe COPD patients were hyperoxic during the first 3 h after arrival at the ED. Almost none were hypoxic and at T_2_ most patients were in the normal range. After being admitted to the nursing ward or ICU, more than half of the severe COPD patients (69%) stayed hyperoxic.

## Discussion

To our knowledge, this study is the first to investigate the feasibility of aiming for normoxia in critically ill patients at the ED. A study performed in the prehospital setting was stopped early because it appeared that it was not feasible to titrate prehospital oxygen therapy to normoxia [7]. We found that it is feasible to titrate oxygen therapy aiming for normoxia in critically ill patients at the ED; normoxia was obtained in 86,4% of the patients within 1 h after arrival at the ED. Furthermore, we found that patients for whom the protocol was not followed were mostly severe COPD (GOLD III/IV) patients (*P* < 0.001), patients with an COPD exacerbation (*P* = 0.03) or other pulmonary conditions (*P* = 0.04). The clinical impression the COPD patient made in the ED appeared to influence which saturation was aimed for by the responsible physician, making it difficult to attain the specific target saturations prescribed by our protocol. Future research will need to validate the ideal target saturation for patients with and without risk of hypercapnic respiratory failure. Despite this, in our opinion, COPD patients could also be treated with titrated oxygen therapy. Of the patients who received prehospital oxygen, patients arriving with a NRM were significantly more often hyperoxic (*P* < 0.001) on arrival (T_0_) compared to patients arriving with a nasal cannula.

Liberal oxygen use is still common. There is still uncertainty about the ideal target saturation, mostly due to lack of evidence from clinical trials [1]. The majority of studies evaluating aiming for normoxia were performed at the ICU. Most ICU clinicians acknowledge the potential adverse effects of hyperoxia and declare low tolerance for high oxygen levels, but in clinical practice a substantial part of their patients were exposed to high arterial oxygen levels [9]. Another ICU study found that hyperoxia was frequently observed but not resulted in adjustment of the ventilator settings [6]. This study’s main strength is that it is a prospective cohort study with inclusion of ED patients with different backgrounds. A limitation was that permission from the neurology department could not be obtained. There are several studies evaluating hyperoxia in neurology patients, however we could not include this subgroup in our study [10–15]. Education and feedback is needed to create more awareness of the importance of aiming for normoxia and applying this in clinical practice. Following the validation of the ideal target saturation, new guidelines for emergency oxygen therapy should be developed. To further assess primary and secondary outcome parameters, multicenter trials are required. Aiming for normoxia immediately after arrival in the hospital could prevent the potential harmful effects of hyperoxia, this could lead to health gains on short- and long term and reduced costs during hospital admission [16].

## Conclusion

In this prospective cohort study we showed that it is feasible to attain normoxia in critically ill patients at the ED, demonstrating that it is possible to start titration of oxygen therapy immediately after arrival at the hospital. These study results can be a starting point for further research assessing the potential beneficial effects of normoxia compared to hyper- or hypoxia in the emergency department and the subsequent development of guidelines.

## Abbreviations

COPD: Chronic obstructive pulmonary disease
ED: Emergency department
ICU: Intensive care unit
NRM: Non-rebreather mask
UMCG: University Medical Center Groningen
VM: VentiMask
